# STRATEGIES OF LIFESPAN REGULATION WITHIN AND ACROSS SPECIES

**DOI:** 10.1093/geroni/igad104.1425

**Published:** 2023-12-21

**Authors:** Vadim Gladyshev

**Affiliations:** Harvard Medical School, Boston, Massachusetts, United States

## Abstract

Lifespan varies within and across species, but the general principles of its control remain unclear. We conducted multi-tissue RNA-seq analyses across mammalian species, identifying longevity signatures and examining their relationship with transcriptomic biomarkers of aging and established lifespan-extending interventions. Signatures of long-lived species were positively correlated with age-related changes and enriched for evolutionarily ancient essential genes. Conversely, lifespan-extending interventions counteracted aging patterns and affected younger, mutable genes enriched for energy metabolism. The identified biomarkers revealed new longevity interventions. We further developed a transcriptomic clock that quantifies progression through lifespan and reports the effects of longevity interventions. Overall, this work uncovers universal and distinct strategies of lifespan regulation within and across species and provides tools for quantifying aging and discovering longevity interventions.

